# Ethical dilemmas in contemporary psychiatry: Findings from a survey of National Psychiatric Associations in Europe

**DOI:** 10.1192/j.eurpsy.2023.2470

**Published:** 2023-11-01

**Authors:** Jerzy Samochowiec, Dorota Frydecka, Karolina Skonieczna-Żydecka, Meryam Schouler-Ocak, Bernardo Carpinello, Eka Chkonia, Geert Dom, Peter Falkai, Błażej Misiak, Mariana Pinto da Costa, Jan Wise, Livia de Picker, Simavi Vahip, Danuta Wasserman, Silvana Galderisi, Przemysław Bieńkowski

**Affiliations:** 1 Department of Psychiatry, Pomeranian Medical University, Szczecin, Poland; 2 Department of Psychiatry, Wroclaw Medical University, Wrocław, Poland; 3 Department of Biochemical Science, Pomeranian Medical University, Szczecin, Poland; 4 Psychiatrische Universitätsklinik der Charité Im St. Hedwig Krankenhaus, Berlin, Germany; 5 Section of Psychiatry, Department of Medical Sciences and Public Health, University of Cagliari, Cagliari, Italy; 6 Department of Psychiatry, Tbilisi State Medical University, Tbilisi, Georgia; 7 Collaborative Antwerp Psychiatric Research Institute (CAPRI), University of Antwerp, Antwerp, Belgium; 8 Department of Psychiatry and Psychotherapy, University Hospital, LMU Munich, München, Germany; 9 Institute of Psychiatry, Psychology & Neuroscience, King’s College London, London, United Kingdom; 10 Adult General Psychiatry CNWL NHS Foundation Trust, London, United Kingdom; 11 University Psychiatric Hospital Campus Duffel, Duffel, Belgium; 12 Department of Psychiatry, Ege University Medicine Faculty, Affective Disorders Unit, Izmir, Turkey; 13 National Centre for Suicide Research and Prevention of Mental Ill-Health, Karolinska Universitet, Stockholm, Sweden; 14 Department of Psychiatry, University of Campania “Luigi Vanvitelli”, Naples, Italy; 15 Department of Psychiatry, Warsaw Medical University, Warsaw, Poland

**Keywords:** ethical codes, ethics, human rights, psychiatry

## Abstract

**Background:**

The European Psychiatric Association (EPA) is an organization that speaks on behalf of its individual members and members of National Psychiatric Associations (NPAs). The aim of this study to identify and investigate current contents of ethical codes and practices in the countries belonging to EPA.

**Methods:**

The study is an expert survey sent out to 44 representatives of 30 NPAs covering the following topics: the existence of national bodies dealing with ethical issues in psychiatry, the availability of documents relevant to ethical issues, the types of ethical issues addressed at the national level, and the current and envisaged ethical debates.

**Results:**

Out of 44 experts invited to participate in the study, 31 NPAs from 30 countries responded (response rate 70.45%). In the majority of countries, the general mission statement serves as the main document covering ethical issues in psychiatry. Most frequently, internal documents were reported to address medical malpractice, workplace bullying, plagiarism, academic fraud, sexual abuse, and discrimination/racism. Furthermore, internal documents cover the ethical assessment of potentially controversial procedures, including psychosurgery, euthanasia, and pregnancy termination. The most important topics for debate at the level of NPAs/EPA were associated with violations of clinical practice standards and human rights.

**Conclusions:**

NPAs are active in the field of professional ethics, defining ethical standards related to interactions among professionals and services provided by mental health care professionals. Future collaboration of NPAs, under the umbrella of the EPA, could allow to develop a database of local ethical documents that would be translated into English and accessible to all EPA members.

## Introduction

It has been forecasted that almost one-third of the global population will experience a mental disorder during their lifetime [1]. Apart from high prevalence rates, psychiatric disorders are associated with stigma, myths, and distorted perceptions, which altogether increase the burden for affected individuals, and their families [2]. The clinical manifestation of several psychiatric disorders often leads to disability, contributing to communication difficulties and impaired social functioning [3]. Exacerbation of psychiatric symptoms may be associated with a risk of harm to self and others, or impaired decision-making, thus leading to the need for inpatient treatment, which requires taking into account country-specific regulations on involuntary admissions and the use of coercive measures [4]. Moreover, symptoms of mental disorders might emerge in the context of social disadvantage or illegal activity, which always need to be considered in light of confidentiality issues. All such challenges that are often faced in routine clinical practice require careful ethical considerations. They are of particular importance for professionals involved in psychiatric care, whose decisional capacity might be influenced by political and/or societal pressures [5].

The term “ethics” captures a variety of moral standards and values held by an individual within themselves [6]. Ethical principles are a crucial foundation of the medical profession. Psychiatric practice, however, differs from other medical fields, and the uniqueness of mental health care setting gives rise to its own distinctive ethical dilemmas. Therefore, it is widely agreed that psychiatry calls for codes of ethics, which go beyond those provided by the bioethical principles applicable to other medical fields [7]. The purpose of developing ethical codes is to serve educational and regulatory functions as well as provide explicit and visible sets of standards for professional practices [8]. The codes developed by international and national associations generally take the form of guiding principles about duties and rights, rather than strict and explicit rules [9].

The necessity to develop specific ethical regulations for psychiatry was formally noted by the American Psychiatric Association (APA) as the consequence of malpractices observed in the 1960s. Indeed, the APA developed the first code of ethics for its members in 1973. However, as the history of psychiatry has shown, malpractice is not the only ethical problem that such codes should address. Hence, the European Psychiatric Association (EPA) published 10 statements referring to a variety of ethical issues related to psychiatric care in 2013^1^. These include (1) promotion of respect for people with mental illness; (2) support of improved quality of psychiatric care; (3) equal access to high-quality care; (4) a call for evidence-based treatment and prevention strategies; (5) promotion of activities against discrimination; (6) support for psychiatric research; (7) protection of confidentiality of information; (8) reduced use of coercive measures; (9) a need for high-standard professional education and (10) regular reporting on implementation progress. The Committee on Ethical Issues of the EPA working under the chairwoman Danuta Wasserman developed the Code of Ethics of the EPA, which was first approved by the Board of the EPA, and thereafter by the EPA General Assembly on April 11, 2021^2^. This and further guidance papers are freely available on the EPA website (www.europsy.net). The EPA Council of National Psychiatric Associations (NPAs) has also addressed ethical aspects of communication in psychiatric practice, by publishing basic and specific principles of communication with respect to informing about the disorder in prodromal stages, recommended treatments, involuntary treatments, communication in case of competing obligations (“dual roles”), genetic counseling, and communication in case of end-of-life conditions [10]. In parallel, the World Psychiatric Association (WPA) developed and released a code of ethics for psychiatry in 2021 [11]. The WPA code of ethics includes four sections: (1) ethics in the clinical practice of psychiatry; (2) ethics in psychiatric education; (3) ethics in psychiatric research and publication, and (4) ethics in psychiatric care.

Although a number of NPAs have produced their own regional/national ethical codes, a recent review of ethical codes within 143 member states of the WPA identified only 15 formal documents [5]. Only a few of them were associated with professional disciplinary processes. Moreover, they were rarely revised against newly emerging challenges for psychiatric care. Indeed, the history of psychiatric care clearly shows that local contexts often affect specific contents of ethical codes [5]. Thus, it is expected that each NPA may differ in terms of the ethical procedures and practices, codes of ethics, and emerging issues to be addressed by revisions of ethical regulations. However, there is a great need for the NPAs to endorse at least the fundamental/overarching ethical principles included in the main international Codes of Ethics, which would be reflected in their national-level equivalent documents. Additionally, in recent years, psychiatry has been facing several new ethical challenges related to the use of novel treatment approaches, evolving concepts of psychiatric genetics, online interventions, palliative care for individuals with mental disorders or the involvement of peer support in psychiatric care [12].

This study originates from the ongoing EPA contribution to the development of ethical codes in psychiatry in the countries of the European Union and beyond. The main objective of this study was to explore expert opinions ethical codes for psychiatry in European countries as well as to inform about the current and future ethical dilemmas that need to be addressed at the level of NPAs and the EPA. To this end, expert representatives of specific NPAs were surveyed to inform about national codes of ethics and emerging ethical dilemmas for psychiatric practice in their respective countries.

## Methods

### Development of the survey

**Figure 1.**
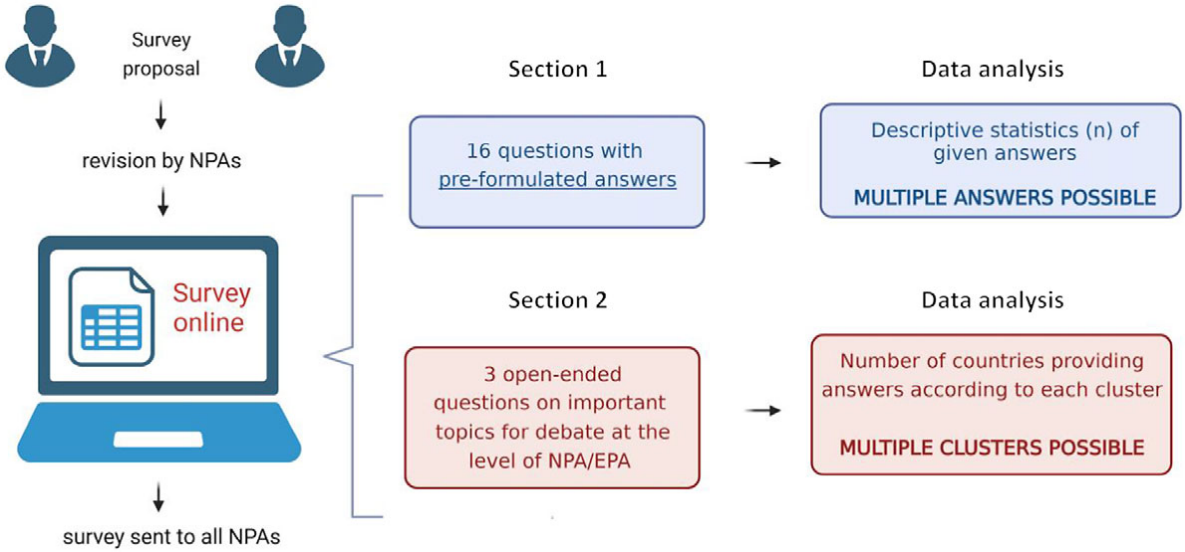
Development of the questionnaire. Abbreviations: EPA, European Psychiatric Association; NPA, National Psychiatric Association; NPAs, National Psychiatric Associations.

The workflow behind the development of the survey is shown in Figure 1. First developed by two co-authors (P.B. and J.S.), the questionnaire was later piloted by NPAs from four countries (Poland, Belgium, Italy, and Turkey). The survey was revised during monthly meetings by three experts (P.B., J.S., and B.M.). The rationale behind developing specific questions was to cover the following aspects of ethics in psychiatry: (1) the presence and characteristics of internal documents related to ethical issues; (2) the presence and characteristics of separate statutory bodies for resolving emerging ethical issues; (3) the presence of governmental bodies on medical ethics developing any recommendations for mental health professionals; (4) implementation of international codes of ethics; (5) education about ethical issues among psychiatric trainees; (6) attitudes towards sharing internal ethical documents and collaboration in developing shared ethical codes; and (7) ethical dilemmas considered the most important to be discussed at the level of NPAs and the EPA.

The final version of the survey is available in the Supplementary Appendix. It was composed of a total of 19 multiple-choice (part I, *n =* 16) and open-ended questions (part II, *n =* 3). The former, that is, part I covered characteristics of existing bodies and documents that address psychiatry-related ethical issues in each surveyed country. Of note, apart from the predefined set of responses, the structure of the survey offered a possibility to provide additional comments. Part II was devoted to ethical dilemmas considered or recognized as paramount for debate at the NPA or NPA/EPA level as well as those anticipated to emerge in the forthcoming years. In this part, multiple answers were possible; however, for each question, there was a possibility to provide a comment on the aspects that were not pre-formulated.

### Participants

Invitations to participate in the survey were sent to all NPAs with a request to indicate the representative with expertise in country-specific documents addressing ethical issues in psychiatry. Altogether, invitations were sent to the boards of 44 NPAs.

### Data collection

The questionnaire was disseminated through the Survey Monkey platform (https://www.surveymonkey.com/r/NPAs). Data were collected until April 2022.

### Data analysis

The survey generated qualitative data. Meetings devoted to the interpretation and operationalization of responses to open-ended questions were held once a month. Due to their diversity, a panel of three independent experts (J.S., P.B., and B.M.) clustered them under the following categories: (1) violations of clinical practice standards (responses: “doctor-patient relationship” and “medical malpractice”); (2) human rights (responses: “compulsory admission”, “informed consent”, “human rights”, “patient’s autonomy versus protection of society”, and “confidentiality”); (3) the role of psychiatrists in making patients’ decisions about euthanasia, or physician-assisted suicide (responses: “assisted suicide/abortion procedures”, “assessment of consciousness”, and “informed consent in specific situations”); (4) stigma/minorities access to care (responses: “psychiatry in general medicine”, “underfunding”, “discrimination”, “private-public interface”, and “access to care including post-COVID care and vaccination”; (5) forensic psychiatry (response: “forensic psychiatry”) and (6) other (responses: “psychiatry and industry”, “workplace bullying”, “cooperation - hospitals versus ambulatory psychiatry”, “private-public interface”, “religion-related aspects of psychiatry”, “reform of psychiatric care”, and “climate”).

Of note, given that not only medical doctors can apply for a membership in many NPAs, in our analysis we decided to use a more general and inclusive term “patient-member relationship” to refer to a range of ethical issues that are normally associated with the “doctor-patient relationship” (e.g., medical malpractice, discrimination, sexual abuse, etc.). The answers given by NPA representatives were presented as numbers and analyzed in a descriptive way. The Excel datasheet was used to perform all calculations.

## Results

We received responses from the representatives of NPAs in thirty countries, including Armenia, Azerbaijan, Belarus, Belgium, Bosnia and Herzegovina, Bulgaria, Croatia, the Czech Republic, Denmark, Estonia, France, Georgia, Germany, Hungary, Israel, Italy, Latvia, Lithuania, Moldavia, Norway, Poland, Portugal, Romania, Russia, Serbia, Slovakia, Switzerland, Turkey, the United Kingdom, and Ukraine. Responses to the survey were obtained from 31 NPA representatives (i.e., 1 per country; with 2 representatives from Russia). Despite multiple attempts, we did not receive any feedback from Austria, Belgium, Finland, Greece, Iceland, Malta, Netherlands, Slovenia, Spain, and Sweden.

In the majority of the surveyed countries (*n =* 12), the main document to address ethical issues in psychiatry is the general mission statement, that is, a brief text presenting the major aims of the NPAs, groups they serve, and the values they follow. Others have a separate ethical code (*n =* 8) or a separate chapter in the associations statute (*n =* 4). The representatives of 4 NPAs (Belgium, France, Lithuania, and Switzerland) reported to have no such documents in their countries, whereas five further ones did not provide any response. A separate statutory body (i.e., committee, working group, section) for resolving emerging ethical issues exists in 21 NPAs.

Out of all ethical issues specifically addressed by internal documents regarding the relationship among medical professionals, medical malpractice was the most frequently reported (*n =* 18), followed by workplace bullying (*n =* 9), plagiarism and academic fraud (*n =* 6), and sexual abuse (*n =* 4). With respect to patient–member relationship, the majority of ethical issues were related to medical malpractice (*n =* 17) and discrimination/racism (*n =* 13). Seven responses of NPA representatives indicated that none of these issues were addressed by internal documents. Ethical assessments of potentially controversial procedures were reported to be present in some internal documents (*n =* 11), and they were relevant to psychosurgery (*n =* 4), euthanasia (*n =* 5), and pregnancy termination (*n =* 2).

Governmental bodies on medical ethics were reported to exist in 27 countries. In 25, psychiatry is represented in these by contracting external independent experts (*n =* 15) or engaging the NPA member (*n =* 7). Governmental bodies on medical ethics were indicated to develop guidelines and viewpoints on mental health issues in 15 countries. The obligation to obtain ethical approval for research in the domain of mental health care was reported to be necessary in almost all countries (*n =* 28). Regarding the respondents’ opinion on the primary source of ethical principles for the NPA members, both international (*n =* 28) and internal (*n =* 22) codes and regulations were indicated. As for the formally adopted international ethical codes, those included the Declaration of Helsinki (*n =* 11) [13], the WPA Declaration of Madrid (*n =* 11) [14], the EPA code (*n =* 6) [15], and the Hawaii declaration (*n =* 1) [16]. We received no response from 6 representatives, and 2 others reported no formal adaptation, instead ethical principles were reported to be disseminated among the members in a different form. Most NPAs accepted the idea of sharing their ethical documents with other NPAs (*n =* 20) to build a database of ethical guidelines supported by the EPA (*n =* 25). Detailed responses to close-ended questions (Q1–Q17) are provided in Table 1.

**Table 1.** Response characteristics to the first part of the survey (multiple-choice questions)

Question	Response range	Responses provided (n)	Countries
Q1. What internal documents does your association have that address ethical issues specific for psychiatry?	none	4	CH, BE, FR,LT
	general mission statement	12	PT, GE, UA, RS, RO, NO, EE, CZ, BiH, MD, TR, DK
	separate chapter in the association’s statute	4	LT, RU, HR, DK
	separate ethical code(s)	8	PL, RU, AM, HU, BY, AZ, UK, TR
Q2. What kind of ethical issues are specifically addressed in your internal documents for member-member relationships?	none	9	CH, PT, BE, UA, RS, FR, HR, CZ, SK
	workplace bullying	7	LT, PL, HU, IL, RU, UK, TR
	medical malpractice	18	LT, GE, IT, PL, RY, AM, HU, BG, IL, RO, NO, EE, BY, AZ, LT, BiH, UK, TR
	sexual abuse	4	HU, IL, UK, TR
	plagiarism/academic fraud	6	IL, RO, BiH, MD, UK
Q3. What kind of ethical issues are specifically addressed in your internal documents for patient-member relationships?	none	8	PT, BE, RS, FR, EE, HR, CZ, MD
	medical malpractice	17	LT, GE, PL, RU (x2), AM, HU, BG, RO, NO, NO, BY, SK, AZ, LT, BiH, UK, TR
	malpractice in psychotherapy	9	PL, AM, HU, BG, RO, RU, AZ, UK, TR
	sexual abuse	8	CH, PL, HU, IL, NO, AZ, UK, TR
	labor abuse	2	HU, IL
	discrimination and racism	13	GE, PL, AM, HU, BG, IL, RU, BY, AZ, BiH, UK, TR, DE
Q4. What kind of ethical issues are specifically addressed in your internal documents for ethical assessment of potentially controversial procedures?	None	20	PT, GE, BE, UA, RS, AM, HU, BG, RO, FR, EE, RU, HR, CZ, BY, SK, LT, MD, UK, TR
	psychosurgery	4	CH, PL, AZ, BiH
	pregnancy termination	2	IT, IL
	euthanasia, physician assisted suicide	5	LT, CH, NO, AZ, DE
Q5. Do you have a separate statutory body (committee, working group, section) for resolving emerging ethical issues?	Yes	21	LT, IT, PL, UA, RU(x2), AM, HU, BG, IL, NO, HR, CZ, BY, SK, AZ, BiH, UK, TR, DE, DK
	No	10	CH, PT, GE, BE, RS, RO, FR, EE, LT, MD
Q6. Is there a governmental body(ies) on medical ethics in your country?	Yes	27	LT, CH, PT, GE, IT, BE, PL, UA, RU(x2), RS, AM, HU, BG, RO, NO, FR, EE, HR, CZ, BY, LT, BIH, UK, TR, DE, DK
	No	4	IL, SK, AZ, MD
Q7. Is psychiatry represented in the above body(ies) dealing with medical ethics?	Yes	25	LT, CH, PT, GE, BE, PL, UA, RU(x2), RS, HU,BG, IL, RO, NO, FR, HR, BY, AZ, LT, BIH, UK, TR, DE, DK
	No	6	IT, AM, EE, CZ, SK, MD
Q8. If you answered yes to the previous question, please indicate how psychiatry is represented in this body(ies)?	via your association	7	CH, GE, RU, AM, AZ, UK, DE
	through recruiting independent experts	15	CH, PT, GE, IT, BE, PL, UA, RS, BG, NO, HR, UK, TR, DE, DK
Q9. Have the governmental bodies on medical ethics in your country developed any guidelines, viewpoints on mental health issues?	Yes	15	CH, PT, IT, BE, PL, UA, RU, BG, IL, NO, HR, LT, UK, DE, DK
	No	16	LT, GE, RS, AM, HU, RO, FR, EE, RU, CZ, BY, SK, AZ, BiH, MD, TR
Q10. For research in mental health care in your country, please indicate if there is an obligation to obtain the ethical vote and if so, which ethical committee is responsible for this vote	Yes	31^a^/	ALL
	No	0	
Q11. In your opinion, the primary source of ethical principles for members of your NPA should be	internal codes and regulations	22	CH, IT, BE, PL, UA, RU (X2), AM, BG, IL, RO, FR, HR, CZ, BY, SK, LT, MD, UK, TR, DE, DK
	international codes and regulations	28	LT, CH, PT, GE, IT, BE, PL, UA, RU (x2), RS, AM, HU, BG, RO, NO, EE, HR, CZ, BY, SK, AZ, LT, BiH, MD, UK, TR, DE
Q12. Has your NPA formally adopted any international ethical codes?	Yes	15	GE, IT, PL, UA, RU (x2), BG, IL, RO, NO, SK, AZ, BiH, UK, DK
	No	16	LT, GE, RS, AM, HU, RO, FR, EE, RU, CZ, BY, SK, AZ, BiH, MD, TR
Q13. If you answered yes to the previous question, please specify which international ethical codes have been adopted	EPA code	6	PT, IT, PL, NO, MD, DK
	WPA Declaration of Madrid	11	GE, UA, RU(x2), NO, EE, AZ, BiH, MD, UK, DK
	Declaration of Helsinki	11	RU, RS, BG, IL, RO, NO, EE, SK, BiH, MD, DK
Q14. Is the topic of ethics included in professional training of young psychiatrists in your country?	Yes	19	CH, IT, BE, PL, UA, RU(x2), RS, AM, BG, IL, RO, NO, EE, HR, BY, LT, TR, DE
	No	12	LT, PT, GE, HU, FR, CZ, SK, AZ, BiH, MD, UK, DK
Q15. Would you be interested in sharing your NPA’s internal documents addressing ethical issues with other NPAs?	Yes	20	PT, IT, BE, PL, RU(x2), RS, AM, BG, RO, NO, FR, HR, CZ, BY, BiH, UK, TR, DE, DK
	No	11	LT, CH, GE, UA, HU, IL, EE, SK, AZ, LT, MD
Q16. Would you be interested in sharing your NPA’s internal ethical documents to create an EPA-supported database available to all NPAs?	Yes	25	CH, PT, IT, BE, PL, RU(x2), RS, AM, HU, BG, RO, NO, FR, HR, CZ, BY, AZ, LT, BiH, MD, UK, TR, DE, DK
	No	6	LT, GE, UA, IL, EE, SK

*n =n =n =*Abbreviations: EPA, the European Psychiatric Association; NPA, National Psychiatric Association; NPAs, National Psychiatric Associations; WPA, the World Psychiatric Association; LT, Lithuania; CH, Switzerland; PT, Portugal; GE, Georgia; IT, Italy; BE, Belgium; PL, Poland; UK, Ukraine; RU, Russia; UA, Ukraine; RS, Serbia; AM, Armenia, HU, Hungary; BG, Bulgaria; IL, Israel; RO, Romania; NO, Norway; FR, France; EE, Estonia, HR, Croatia; CZ, Czech; BY, Belarus; SK, Slovakia; AZ, Azerbaijan; LT, Lithuania; BiH, Bosnia and Hercegovina; MD, Moldova; UK, United Kingdom; TR, Turkey; DE, Germany; DK, Denmark.

a no answer: *n =* 1; good practice but no obligation: *n =* 1; only if research includes patient identifiable information: *n =* 1.

Table 2 presents responses to the second part of the survey, including questions on the ethical dilemmas considered or recognized as essential for debate at the NPA or NPA/EPA level as well as ones anticipated to emerge in the forthcoming years. The most important topics for current debate at the NPA level were associated with violations of clinical practice standards and human rights, whereas the ethical dilemmas recognized as the most important for debate at the NPA/EPA level in the upcoming years were associated with human rights.

**Table 2.** Response characteristics to the second part of the survey (open-ended questions)

Question	Responses		Countries providing YES answers
	Yes (n)	No (n)	
Q17. What ethical dilemmas do you consider the most important topic for debate in your NPA? Category 1: Violations of clinical practice standards Category 2: Human rights Category 3: The role of psychiatrists in making patient decisions about euthanasia, or physician-assisted suicide Category 4: Stigma/minorities access to care Category 5: Forensic psychiatry Category 6: Other			
	10	21	GE, IT, UA, RS, BG, EE, CZ BiH, MD, LA
	13	18	RO, FR, RU, HR, CZ, BY, SK, AZ, LT, TR, DE, CH, PT
	7	24	BE, PL, IS, FR, HR, DE, CH
	7	24	PL, AM, HU, BG, FR, UK, DK
	1	30	RU
	5	26	GE, PL, RU, NO, BiH
Q18. What ethical dilemmas do you recognize as the most important topic for debate at the level of NPA/EPA? Category 1: Violations of clinical practice standards Category 2: Human rights Category 3: The role of psychiatrists in making patient decisions about euthanasia, or physician-assisted suicide Category 4: Stigma/minorities access to care Category 5: Forensic psychiatry Category 6: Other			
	7	24	GE, UA, RU, RS, CZ, LA, PT
	12	19	IT, BE, CZ, BY, AZ, LT, BiH, TR, DE, DK, CH, PT
	8	23	IT, BE, RO, EE, HR, BiH, DE, CH
	8	23	BE, AM, HU, BG, HR, BiH, UK, DK
	0	31	
	6	25	GE, PL, RU, NO, RU, SK
Q19. What ethical dilemmas do you foresee as the most important topic for debate in the years to come? Category 1: Violations of clinical practice standards Category 2: Human rights Category 3: The role of psychiatrists in making patient decisions about euthanasia, or physician-assisted suicide Category 4: Stigma/minorities access to care Category 5: Forensic psychiatry Category 6: Other			
	6	25	RS, BG, SK, AZ, LA, PT
	14	17	IT, BE, PL, FR, CZ, SK, LT, BiH, MD, UK, TR, DE, CH, PT
	6	25	IT, BE, PL, HR, DE, CH
	10	21	BE, AM, HU, BG, IS, NO, RU, HR, BY, DK
	0	31	
	4	27	GE, RU, RO, NO

Abbreviations: EPA, the European Psychiatric Association; NPA, National Psychiatric Association; NPAs, National Psychiatric Associations; LT, Lithuania; CH, Switzerland; PT, Portugal; GE, Georgia; IT, Italy; BE, Belgium; PL, Poland; UK, Ukraine; RU, Russia; UA, Ukraine; RS, Serbia; AM, Armenia; HU, Hungary; BG, Bulgaria; IL, Israel; RO, Romania; NO, Norway; FR, France; EE, Estonia; HR, Croatia; CZ, Czech; BY, Belarus; SK, Slovakia; AZ, Azerbaijan; LT, Lithuania; BiH, Bosnia and Hercegovina; MD, Moldova; UK, United Kingdom; TR, Turkey; DE, Germany; DK, Denmark.

## Discussion

### Main findings

In our study, we explored practices of European NPAs and procedures addressing ethical issues specific to psychiatry and related disciplines. NPAs play an active role in establishing ethical guidelines for psychiatrists in their respective countries. They have either general mission statements, separate ethical codes in the form of internal documents or separate chapters in their individual statutes to address ethical issues that are specific for psychiatry. Although they cover ethical directions for psychiatric professionals in a very limited and brief form, and should therefore be substituted by more detailed codes of conduct in future, general mission statements prove to serve as the main documents to address ethical issues in psychiatry by the majority of the countries that took part in the survey (18 out of 31). Only a minority of NPAs (8 out of 31) turned out to have their own ethical codes. Separate ethical codes were developed by the Armenian, Russian, Hungarian, Belarussian, Turkish, Polish, and Azerbaijan psychiatric associations as well as the Royal College of Psychiatrists in the UK. Having a separate chapter in their association’s statute was reported by 4 NPAs: Danish, Croatian, Russian, and Latvian. The low number of NPAs with separate ethical codes or separate chapters in their statute remains in line with reports of Bloch et al. [5], who found that out of 143 member societies of the WPA, only 15 organizations had their own ethical codes, of mostly hybrid nature, containing both morally based principles and explicit rules typical for codes of conduct and clinical guidelines.

International and local ethical codes addressing ethical dilemmas specific to psychiatric care can be seen as central to the ethical reflection on contemporary psychiatry. Our findings show that such documents cover ethical issues concerning member-member and patient-member relationships, including medical malpractice, workplace bullying, plagiarism and academic fraud, sexual abuse, and discrimination/racism. Additionally, available documents for ethical assessment address several potentially controversial procedures, including psychosurgery, euthanasia, and termination of pregnancy. Out of these, the issues related to medical malpractice, discrimination, and racism tend to stand out. It is not particularly surprising as these or similar issues constitute a core of the widely recognized international ethical codes. Hence, it is possible that for some NPAs, local ethical codes may be viewed as unnecessary. Further support for this hypothesis comes from the observation that in the majority of member countries, there is a governmental body dealing with medical ethics in psychiatry, where psychiatric expertise is represented by an external expert, or the NPA member (as reported by 25 out of 31 NPA representatives), and in almost half of the surveyed countries (15 out of 31), governmental bodies on medical ethics developed guidelines and viewpoints on mental health issues.

According to the vast majority of NPA representatives (28 out of 31 representatives), the primary source of ethical principles should be adopted from and in line with international ethical codes and regulations. To date, there are 15 countries that formally adopted internationally recognized ethical codes, such as the Declaration of Helsinki [13], the WPA Declaration of Madrid [14], the EPA Declaration on Quality of Psychiatry and Mental Health Care in Europe [15], and the Declaration of Hawaii [16]. Additionally, most NPA representatives agreed to share their ethical documents with other NPAs to build a joint database of ethical guidelines supported by the EPA; however, the EPA Code is still to be adopted by most of the NPAs in Europe as at the time of the survey only six of them. It is especially important since the existence of universal ethical codes could provide an important common language and standards that may guide ethical behavior internationally despite political imperatives [8]. It is striking that the same number of NPAs (11 out of 31) declared a formal approval of the Declaration of Helsinki [13] and the Declaration of Madrid [14]. The popularity of the Declaration of Helsinki may result from the fact that many boards of NPAs are dominated by research-oriented individuals, who apply medical ethics to their academic and industry-sponsored research. Given the research-related content of the Declaration of Helsinki and a rapid progress of psychiatric sciences, one would expect a solid representation of research-related ethical dilemmas among the answers to the open-ended questions. Contrary to our expectations, however, this was not the case as issues related to human rights as well as stigmatization and discrimination dominated topics selected for current and future debate.

Importantly, findings from this survey indicate that human rights and violations of clinical practice are perceived by the majority of NPAs as the most important issues for debate in the upcoming years. Such considerations might be of importance for emerging ethical challenges related to implementation of artificial intelligence, precision psychiatry, telepsychiatry, and the lack of evidence-based approaches in the real-world clinical practice. Artificial intelligence refers to a range of computer-based algorithms that show abilities heretofore attributable to “human intelligence” [17]. From the perspective of precision psychiatry, the far goal of implementing artificial intelligence would be to improve decision-making in terms of risk assessment and personalization of treatment. To date, a variety of ethical issues related to the use of artificial intelligence in psychiatric practice have been identified and include, e.g., communication of incidental findings, consequences for the self-image of people with mental disorders and health care professionals as autonomous individuals, biased and poorly generalizable predictions, inability to handle the complexity of social contexts and problems with data protection [18–20]. Notably, a variety of ethical issues related to artificial intelligence can be resolved through the general principles of ethics in biomedicine (e.g., beneficence, non-maleficence, or respect for individual autonomy) and communication [18]. However, certain aspects related to transparency and the possibility to explain responsibility for specific predictions, especially in case of unfavorable or undesirable outcomes, might appear to be problematic. As similar to artificial intelligence, telepsychiatry can use the general principles of ethics related to confidentiality, communication, consent, or data protection. Nevertheless, several challenges for ethical issues related to telepsychiatry still exist and include depersonalization of doctor–patient relationship, the need to develop cross-border legislations, the extent of data storage and recording, and the adaptation of consent to novel technologies [21]. Finally, the debate on violations of clinical practice will need to recognize the real-world utility of practice guidelines and determinants of the evidence-practice gap in psychiatry. For instance, a recent systematic review and meta-analysis revealed that guideline implementation does not impact provider performance but may still positively influence patient outcomes [22].

### Strengths and limitations

To the best of our knowledge, this is the first survey on ethical practices used by NPAs in Europe. The survey content was prepared by the Working Group of the NPAs Committee as well as officially reviewed and approved by the EPA statutory bodies. Individual NPA boards were approached repeatedly via the NPA Committee and the response rate was quite high (31 out of 44 NPAs approached for participation, 70.4%). Nevertheless, this study is not free from certain limitations, which need to be considered. First of all, our results may rather reflect the opinions of prominent members of NPA statutory bodies than views (or real-life practices) shared by representative samples of healthcare professionals. What is more, the meaning and significance of documents and statutory bodies dealing with ethical dilemmas as well as the role played by the NPAs in the local psychiatric communities (e.g., the college of professionals versus the scientific association) may differ from country to country. Finally, although relatively high, the response rate does not allow to generalize the results of the survey to all NPAs associated with the umbrella of the EPA.

### Implications of the findings for future practice and research

In the future, studies on local or national ethical documents could analyze medical, social, and political factors behind their development. It would also seem worthwhile to identify cases in which ethical codes were prepared in a retroactive manner, that is, as a direct response to cases of unethical conduct of psychiatrists or psychotherapists. This survey was not intended to compare or differentiate between local ethical codes, and it remains an open question as to whether ethical documents developed by individual NPAs contain more general moral concepts or explicit rules for clinical practice. The results of our study could be a starting point for future in-depth comparative research on ethical perspectives represented in National Psychiatric Associations across Europe. Lastly, it should be underlined that empirical studies are needed to measure ethical codes’ awareness and their effectiveness in influencing professional conduct. In addition, no information was provided of the actual implementation (and associated procedures) of national/regional codes.

## Conclusions

The NPAs seem to be active in the field of professional ethics but prefer to rely on international ethical codes rather than develop their own ethical guidelines. Given the declared openness of the NPAs to share their internal ethical documents, their future collaborative activities under the umbrella of the EPA could result in developing a joint database thereof a resolution to address emerging ethical dilemmas. Nevertheless, a debate about joint resolutions will be called for as differences in country-specific laws and ethical regulations need to be considered. Future collaboration of NPAs, under the umbrella of the EPA, could allow to develop a database of local ethical documents that would be translated into English and accessible to all EPA members. Prospective steps should include further international debates resulting in standardization of practices across Europe and joint resolutions for emerging ethical dilemmas in psychiatry.

## Supporting information

Samochowiec et al. supplementary material Samochowiec et al. supplementary material

Samochowiec et al. supplementary material Samochowiec et al. supplementary material

## Footnotes

1
https://www.europsy.net/app/uploads/2014/07/Declaration_for-publication.pdf.

2
https://www.europsy.net/app/uploads/2021/06/EPA-Code-of-Ethics_March-2021-GA-approved.pdf.

## Supplementary material

The supplementary material for this article can be found at http://doi.org/10.1192/j.eurpsy.2023.2470.
