# Supplementary material for: Ethical dilemmas in contemporary psychiatry: Findings from a survey of National Psychiatric Associations in Europe
Source: Eur Psychiatry. 2023 Nov 1;66(1):e94. doi: 10.1192/j.eurpsy.2023.2470 (PMC10755573; doi:10.1192/j.eurpsy.2023.2470)
Supplement: Samochowiec et al. supplementary material [file S0924933823024707sup001.docx]

**Supplementary Appendix**

**The survey used in the present study**

***Scope:***

- compare NPA's procedures and practices addressing ethical issues specific to psychiatry and related disciplines
- assess recognition and implementation of international ethical codes
- identify emerging ethical issues to be addressed in future EPA/NPA activities
- promote building of EPA/NPA-supported database of ethical codes and practices existing in European psychiatry
- facilitate discussion and exchange of best practices between NPAs

***Dear Colleagues,***

Although ethical principles constitute a core aspect of clinical medicine, including psychiatry, we all face various ethical dilemmas in routine psychiatric care and in psychiatric research.

Dealing with ethical dilemmas in psychiatry can vary between European countries and regions. Thus, comparing practices in this area could be a good starting point for ongoing discussion and collaboration between NPAs.

In order to facilitate discussion and collaboration, a Task Force group working under the umbrella of the NPA Council prepared a questionnaire gathering information on ethical codes, procedures, and practices used by NPAs. Please share with us your procedures and practices by filling the questionnaire below.

1. What internal documents does your association have that address ethical issues specific for psychiatry? (Tick one or more boxes as appropriate)

- none
- general mission statement
- separate chapter in the association's statute
- separate ethical code(s)
- other (please specify)

1. What kind of ethical issues are specifically addressed in your internal documents for member-member relationships? (Tick one or more boxes as appropriate)

- workplace bullying
- medical malpractice
- sexual abuse
- plagiarism, academic fraud
- other (please specify)
- none

1. What kind of ethical issues are specifically addressed in your internal documents for patient-member relationships? (Tick one or more boxes as appropriate)

- medical malpractice
- malpractice in psychotherapy
- sexual abuse
- labour abuse
- discrimination/racism
- other (please specify)
- none

1. What kind of ethical issues are specifically addressed in your internal documents for ethical assessment of potentially controversial procedures? (Tick one or more boxes as appropriate)

- psychosurgery
- pregnancy termination
- euthanasia/PAS (Physician assisted suicide)
- other (please specify)
- none

1. Do you have a separate statutory body (committee, working group, section) for resolving emerging ethical issues?

- No
- Yes

1. If you answered yes to the previous question, please indicate the name and role of separate statutory bodies (committee, working group, section) working to resolve emerging ethical issues

- Name of body _______________

Major role ________________

- Name of body _______________

Major role ________________

- Name of body _______________

Major role ________________

- Other solutions adopted to resolve emerging ethical issues, please specify _________

1. Is there a governmental body(ies) on medical ethics in your country?

- No
- Yes, please list the governmental body(ies) on medical ethics in your country (professional associations or academic bodies could also be added here)

1. Is psychiatry represented in the above body(ies) dealing with medical ethics?

- No
- Yes

1. If you answered yes to the previous question, please indicate how psychiatry is represented in this body(ies)?

- via your Association
- through recruiting independent experts
- other solutions adopted (please specify)
- psychiatry is not represented in the above body(ies) dealing with medical ethics

1. Have the governmental bodies on medical ethics in your country developed any guidelines, viewpoints on mental health issues?

- No
- Yes, please specify the topic(s) addressed

1. For research in mental health care in your country, please indicate if there is an obligation to obtain the ethical vote and if so, which ethical committee is responsible for this vote.

_________________________

1. In your opinion, the primary source of ethical principles for members of your NPA should be: (Tick one or more boxes as appropriate)

- internal codes and regulations
- international codes and regulations
- please indicate any comment(s) here

1. Has your NPA formally adopted any international ethical codes?

- No
- Yes

1. If you answered yes to the previous question, please specify which international ethical codes have been adopted

- EPA code
- WPA Declaration of Madrid
- Declaration of Helsinki
- Other (please specify)
- My NPA has not formally adopted any international ethical codes

1. Is the topic of ethics included in professional training of young psychiatrists in your country?

- No
- Yes (please specify)

1. Would you be interested in sharing your NPA's internal documents addressing ethical issues with other NPAs?

- No
- Yes

1. Would you be interested in sharing your NPA's internal ethical documents to create an EPA-supported database available to all NPAs?

- No
- Yes

1. What ethical dilemmas do you consider the most important topic for debate in your NPA?

___________________

1. What ethical dilemmas do you recognise as the most important topic for debate at the level of NPA / EPA?

___________________

1. What ethical dilemmas do you foresee as the most important topic for debate in the years to come?

___________________

1. Please indicate your name

___________________

1. Please indicate the name of your NPA

____________________

1. Please indicate your role/position at your NPA

____________________
